# Modelling the Noise-Robustness of Infants’ Word Representations: The Impact of Previous Experience

**DOI:** 10.1371/journal.pone.0132245

**Published:** 2015-07-28

**Authors:** Christina Bergmann, Louis ten Bosch, Paula Fikkert, Lou Boves

**Affiliations:** Centre for Language Studies, Radboud University, Nijmegen, The Netherlands; Birkbeck College, UNITED KINGDOM

## Abstract

During language acquisition, infants frequently encounter ambient noise. We present a computational model to address whether specific acoustic processing abilities are necessary to detect known words in moderate noise—an ability attested experimentally in infants. The model implements a general purpose speech encoding and word detection procedure. Importantly, the model contains no dedicated processes for removing or cancelling out ambient noise, and it can replicate the patterns of results obtained in several infant experiments. In addition to noise, we also addressed the role of previous experience with particular target words: does the frequency of a word matter, and does it play a role whether that word has been spoken by one or multiple speakers? The simulation results show that both factors affect noise robustness. We also investigated how robust word detection is to changes in speaker identity by comparing words spoken by known versus unknown speakers during the simulated test. This factor interacted with both noise level and past experience, showing that an increase in exposure is only helpful when a familiar speaker provides the test material. Added variability proved helpful only when encountering an unknown speaker. Finally, we addressed whether infants need to recognise specific words, or whether a more parsimonious explanation of infant behaviour, which we refer to as matching, is sufficient. Recognition involves a focus of attention on a specific target word, while matching only requires finding the best correspondence of acoustic input to a known pattern in the memory. Attending to a specific target word proves to be more noise robust, but a general word matching procedure can be sufficient to simulate experimental data stemming from young infants. A change from acoustic matching to targeted recognition provides an explanation of the improvements observed in infants around their first birthday. In summary, we present a computational model incorporating only the processes infants might employ when hearing words in noise. Our findings show that a parsimonious interpretation of behaviour is sufficient and we offer a formal account of emerging abilities.

## Introduction

From the moment they are born, and probably even before that, infants are exposed to acoustic signals from a mix of sources, such as a mother speaking to her infant with the television running in the background. Infants hardly ever hear completely noise-free speech [1] and this will have an impact on the language acquisition process. Given the pervasive presence of a somewhat noisy acoustic ‘scene’ in which infants (and adults) are living, relatively little research has been conducted to investigate infants’ speech processing capabilities in noisy environments. Understanding the impact of noise on language acquisition is all the more important because very noisy environments have been linked to a disadvantage in language acquisition [2].

In the present paper, we employ a computational model to study which abilities infants minimally need to bring to the task. Our goal is to explain the overall patterns of performance observed in the handful of experimental studies that exist on infant speech perception in noise. To this end, we first discuss common behavioural methods employed in infant studies, then review existing infant studies and subsequently formulate the goals of this paper. In the model section we describe the background against which we develop our model to clarify why we chose a computational approach.

Experimental research into the cognitive capabilities of infants is far from straightforward; studies have to rely on behavioural responses to external stimulation. Many experiments that investigate early language acquisition are based on procedures that measure *listening preferences*. In a typical instance of a listening preference study, the time infants spend attending to a given speech stimulus is measured. To this end, unrelated visual targets are presented along with sounds. Both the visual target and the sound stop shortly after the infant looks away, thus coupling looking at the target to listening times. When infants’ looking times, and thus presumably listening durations and interest in the acoustic stimuli, diverge systematically across stimulus types (e.g., comparing the infant’s own name to a foil [3, 4]), infants are presumed to process these acoustic stimuli differently. What *processing* means exactly depends on the theories that guide the design of the experiment.

Many infant experiments described in the literature consist of two phases. In the first phase infants are familiarised with a stimulus, for example two words. In the following test phase the responses to new tokens of the same stimulus they previously heard are compared to responses when hearing a completely novel stimulus. The above described set-up can also be used to investigate how infants process words that they might have learned before they come into the lab. In these cases the listening durations in response to putatively known and unknown (but similar) words are compared without prior familiarisation.

Most infant experiments use natural speech that was recorded under ideal conditions. Only a small number of experiments have investigated how infants process speech in the presence of competing speakers or other background noise. Almost all experimental research has found that infants perform substantially worse than adults when they have to process speech in adverse acoustic conditions. More precisely, infants seemingly fail to detect words at noise levels which hardly affect adults, namely in environments comparable to a busy cafè or restaurant. Overall, factors that are relevant for adults in noise seem to affect infants’ abilities in similar ways. It matters, for example, whether the target speaker is familiar or not. It is as yet unclear exactly how infants process speech in noisy environments. Computational modelling, unlike experimental infant studies, offers the possibility to investigate which processes are necessary and sufficient to explain infants’ attested abilities.

One of the first studies that addressed the robustness of infants’ early word representations in the presence of a competing speaker was conducted by Newman and Jusczyk [5]. The authors found that 7.5-month-old infants, after having been familiarised with words which were spoken by a female speaker, could distinguish those words from novel ones, despite the presence of a distracting male voice that was 10 and 5 decibel (dB) less loud (expressed as signal-to-noise ratios, SNRs, of 10 and 5 dB, respectively). This work was extended by Barker and Newman [1] who used female speakers as target and as distractors. They found that infants were only able to solve the task when the words were spoken by a well-known target speaker, i.e., the infant’s own mother. If the target was an unknown female speaker, infants failed to distinguish the familiarised words from novel words, even at an SNR of 10 dB. One explanation for the difference between this result and the findings of Newman and Jusczyk [5] is that it is easier to separate a female voice from a male voice than to separate two female voices. Extending these findings, Hollich and colleagues [6] showed that synchronised information aids 7.5-month-old infants in succeeding at the task of distinguishing familiar from novel words in noise, even at 0 dB SNR. In this work, infants saw videos of a woman who pronounced the familiarisation stimuli along with hearing the target and the competing signal. Only when the visual and the audio stimuli were synchronised could infants harness the additional visual cues.

In a study without a familiarisation phase Newman [4] found that 5- and 9-month-olds can detect their own name, one of the earliest words in their vocabulary [3], in the presence of multi-talker background noise at 10 dB SNR, but not at 5 dB SNR. Around their first birthday infants can detect their name even in noisier conditions at 5 dB SNR. This change in the ability to deal with noise can be due to many factors, ranging from an overall improvement in linguistic processing abilities to a changed strategy when listening to speech in noise. Following up on this study, infants were found to differ from adults at 5 months: When multiple speakers provided the background noise at 10 dB SNR, infants recognized their own name, but not when a single voice competed with the target signal [7]. At 8.5 months, infants can accommodate both single- and multi-talker background noise and detect the presence of their own name compared to matched foils. Adults, in contrast seem to find it easier to separate two voices than separating one target voice from a mix of background talkers. Recent work has exchanged distracting signals from other talkers with uniform noise. In white noise at 10 dB SNR, it plays a role for 8-month-olds whether the spectral channels of the white noise overlap with the target speech or not [8]. This means that infants’ ability to distinguish their name from other names was hampered more by a perceptual overlap which is already present in the first stages of auditory processing (so-called energetic masking), than the presence of a distractor which diverts attention and has to be segregated (informational masking). This finding points to infants being able to focus at least to some extent on the target signal.

Next to target words in the presence of distracting speech or white noise, a second line of inquiry has investigated the processing of sounds while hearing different types of non-speech noise. We present these results briefly for completeness, but focus on the studies discussed above in the remainder of the paper. Polka, Rvachew, and Molnar [9] found that about half of the 6- to 8-month-old infants in their experiment were not able to discover the difference between /bu/ and /gu/ syllables when the speech signals were mixed with cricket noise or bird song during the familiarisation phase. There was no difference between a group of infants that heard the noisy signals both during familiarisation and test and a group that was familiarised with the noisy stimuli and tested with noise-free speech. Contrastingly, of the infants familiarised and tested with noise-free speech all but one succeeded at the task. The mixed speech and background signals were constructed such that there was no overlap in the frequency bands. Therefore, the speech stimuli were not affected by any kind of energetic masking caused by overlapping frequency bands; this leaves some form of informational masking [10] as the most likely explanation for the difficulty encountered by the infants. Comparing these results to the above-mentioned findings on the word-level, it seems that infants’ abilities are disturbed by informational masking, but to a lesser extent than when encountering energetic masking [8].

Countering the effects of energetic and, even more so, informational masking requires some form of *auditory stream segregation* [11]. Stream segregation comes seemingly effortless to adult listeners in moderately noisy conditions: it is usually not too difficult to attend to a conversation partner in a busy restaurant. Adults appear to combine a variety of processes to understand speech in noise. Directional hearing is among the most powerful tools for this purpose, which relies on the signal arriving at different time points at each ear depending on the origin. Attending to the speakers’ lip movements is also helpful. Exactly how stream segregation is accomplished in specific acoustic contexts is not yet completely understood, but it is likely that adults combine strategies based on bottom-up signal processing, such as directional hearing and top-down processing, such as focusing attention on specific aspects of the signal and predicting missing words based on linguistic knowledge [12]. In the infant experiments summarised above the two most powerful processes that can be invoked in stream segregation, observing lip movements and directional hearing, were unavailable. Instead, infants listened to a mix of voices or to one voice and added non-speech noise that was played over a single loudspeaker in the absence of visual cues. Given these restrictions only processes remain that require substantial top-down prediction and active focusing of attention on detailed features of the speech signal to counter the adverse impact of noise. It seems unlikely that infants already posses the ability to predict the speech input given they only know a few words [13].

### Goals of the Present Paper

While there seems to be agreement in the field that infants lack most of the tools that adults employ when comprehending speech in noise, behavioural experiments provide evidence that 6- to 8-month-olds can accommodate speech in noise to some extent, even in the adverse conditions that prevail in infant experiments. Which capabilities that infants can conceivably bring to the task and that do not require sophisticated linguistic abilities can account for the experimental results summarised above? Experimental studies show that infants usually succeed in detecting words in noise of 10 dB SNR, and that they have greater difficulty detecting words 5 dB SNR (depending on the age and the test conditions); success at 0 dB requires the task to be simplified [6]. We thus take the two noise levels of 10 and 5 dB SNR as crucial test case and expect our model to replicate the behavioural pattern emerging from the literature [4, 5], namely relatively robust word detection at 10 dB SNR, and a severe decrease of these abilities at 5 dB SNR.

During the formalisation of the proposed abilities and of infants’ experiences, the following issues became apparent; we thus examine them in addition to word detection in noisy conditions (more precisely, at 10 and 5 dB SNR): first, previous experience might influence infants’ performance and some of the observed variability between participants could stem from differences in what they previously heard outside the lab, especially in studies that relied on previous experience. We target two factors that have previously been proposed to affect infants’ performance when recognising previously learned words, namely word frequency and whether or not multiple speakers uttered this word [4, 5]. Experimental data indicate that multiple voices in the input, opposed to a single voice, can aid infants in building representations that seem more sophisticated [14], and which might be more noise-robust as well. The second issue is the preference for and better performance with familiar voices [1, 15]. We directly test this by simulating the typical test situation where infants hear a completely unknown speaker and contrast this against testing with the speaker that provided the learning material. A third factor pertains to our current lack of knowledge what exactly drives infant behaviour in experimental settings [16]. Is it the recognition of a specific word, which requires attentional focus to that target? Or is any match with previously stored acoustic representation sufficient to generate the observed outcomes? Especially in experiments that did not use the familiarisation-followed-by-test protocol [4, 7, 8] it is tempting to conclude that infants *recognise* their name, i.e., that infants are able to link the acoustic input to some abstract representation and they expect this specific word to occur. However, for the familiarisation-followed-by-test protocol previous work demonstrated that a more parsimonious interpretation is possible [17]: to distinguish between familiar and unknown words, a simple match of purely acoustic representations suffices; abstract or cross-modal cues to the meaning of a word or phrase were not necessary to simulate infant behaviour. We will indicate the reliance on acoustic representations alone without making use of a form of meaning by the term *matching*. Both concepts will be defined in more detail in the Model section.

## The Computational Model

### Ethics Statement

All data reported in the present paper stem from computational modelling studies and did not involve human participants. The speech material used was obtained with the informed consent of all speakers (see Section on Speech Material for further details).

### Background: Computational Models of Infant Language Development

Experiments that test infants in laboratory settings can yield information about capabilities at a certain point in development. However, these tests only provide limited insight into the cognitive processes that underlie the acquisition and improvement of those skills. Because cognitive processes cannot be observed directly, computational simulations provide promising tools for gaining insights into the underlying processes. Most existing computational models have relatively modest goals: they aim to investigate to what extent a learning strategy can succeed in simulating abilities proposed to underlie the behaviours observed in specific (sets of) experiments. For example, models might, like infants, distinguish between two syllables, such as /bu/ and /gu/ [9]; associate monosyllabic non-words (e.g., /lif/ or /neem/) with pictures of different objects [18, 19]; or segment a syllable stream into a sequence of words [20]. Across modelling efforts, it is important to note that no exact behavioural patterns are being replicated, instead model data are compared to trends found in experimental group data, such as success in one condition and failure in another (see e.g., [19]). This results in a tension between experiments measuring skills based on external behaviours at a specific time point and models aiming to address internal processes and general development.

Existing models of word learning share an important characteristic: they all represent auditory stimuli in the form of hand-crafted discrete units, which may be symbols (words or phonemes), or putative sub-symbolic units such as phonetic feature vectors. Further, the input is frequently adapted to the requirements of a given learning algorithm. Thus, existing models assume that there is a black box operating that can convert speech signals into sequences of the type of units that the model takes as input representations [21]. The results of simulations with these models depend as much on the decisions about the input representations as on the learning mechanisms that they propose. Specific input representations make implicit, but crucially important assumptions about infants’ abilities necessary to solve a given task. In the context of the present paper, a further problem arises: the representation of noise. While it is already difficult to construct representations of noise-free speech, creating credible representations of speech in noise is virtually impossible. We thus cannot rely on existing models in the present paper.

The model presented in this paper takes real speech, noise-free as well as noisy, as input and thus sidesteps the problem of hand-crafting credible input. One other model of language acquisition that takes speech as input and links it to cross-modal information is the Cross-channel Early Lexical Learning (CELL) model [22]. In CELL all processes from the raw acoustic signal to the word acquisition and comprehension process are included and made transparent. CELL uses techniques from automatic speech recognition to convert speech signals into a lattice of phone symbols. These techniques require substantial previous learning and can be very sensitive to noise and speaker differences, the crucial factors in the present paper. Thus, we could not employ CELL for the modelling task at hand.

Many computational models of word learning [22] assume some form of multi-modal input, in which speech is accompanied by information that suggests categories; this is akin to supervised learning. Infants learn by trial and error and they receive mostly implicit and sometimes ambiguous feedback, be it external, such as caregiver responses, or internal, for example obtaining a toy, on their actions which provides some level of supervision. In machine learning literature, this corresponds to reinforcement learning. Experiments with infants have shown that learning is most efficient when the feedback is unambiguous [23–25]. For the simulations in this paper we are using unambiguous labels and as a consequence strictly supervised learning. However, it has been shown that a model similar to the one employed in the present paper can also learn when the feedback is not as systematic and error-free [26]. We limited ourselves to unambiguous input in this paper, since a throughout formalisation and investigation of ambiguous feedback requires a more in-depth examination than permitted in the context of this paper. In turn, we only let the model learn from a small amount of speech, amounting to what infants hear over a few days [27], so that it becomes plausible that infants receive a comparable amount of input within their first months paired with clear and unambiguous feedback (see also [28] for a review of supporting findings).

#### A theory of early language development

It is instructive—and for substantial theoretical progress even necessary—to relate computational models of language acquisition to a theory. PRIMIR, a developmental framework for Processing Rich Information from Multidimensional Interactive Representations [29], is at once a functional specification of a comprehensive theory of language acquisition and a reference for interpreting computational models that aim to investigate a specific part of this comprehensive theory. PRIMIR starts from the observation that speech signals carry linguistic, para-linguistic (intonation, stress) and extra-linguistic (speaker identity and gender) information. To acquire the native language a child must pick up and organise the information in the signal along a number of multidimensional interactive planes. The interactions between the representations on these planes are implemented by dynamic filters that help to reorganise the representations during the acquisition process. The lowest level in PRIMIR is the General Perceptual plane that represents the raw speech signal. Importantly, during the first stages of language acquisition, when concepts such as ‘word’, ‘syllable’, or ‘phoneme’ that eventually come to live on higher-level planes are not yet available; what is stored and represented in the General Perceptual plane corresponds to utterances, i.e., stretches of speech separated by clear pauses. Most of the computational models mentioned above can be conceived of as simulating representations and processes that are within PRIMIR confined to planes which emerge later during development. CELL is an exception, in that it aims to simulate the emergence of links between the Perceptual plane and planes that are more specialised, containing information on meaning or conceptual representations. The model that we are going to present next mainly implements the General Perceptual plane, although it shares with CELL that it encompasses links to a plane that represents ‘objects’ which provide supervision for the learning process.

### The Present Model

**Fig 1:**
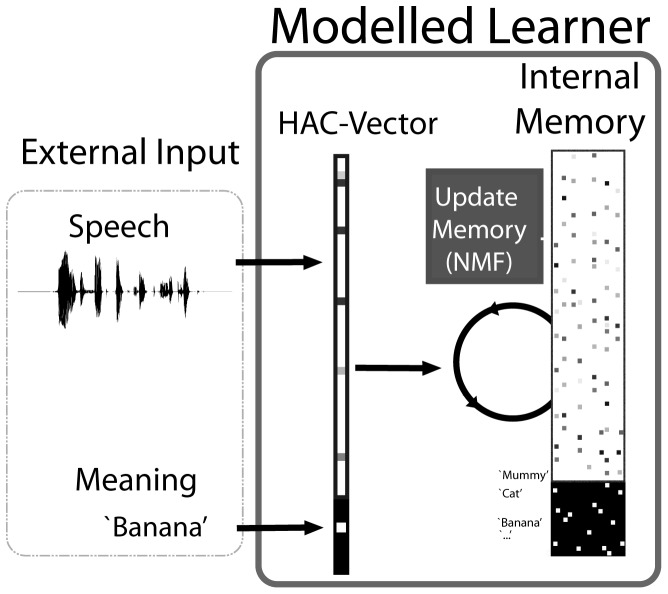
The model in learning mode. Input is presented as speech-meaning pair. After acoustic preprocessing, the memory is adapted to better accommodate the new learning experience.

**Fig 2:**
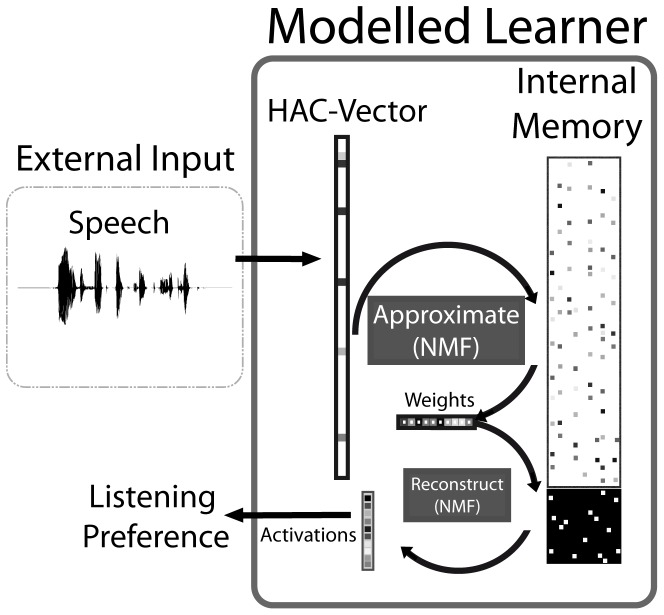
The model in test mode. Input (top left) is presented without meaning information, which has to be reconstructed using the fixed internal memory. The resulting activations are transformed into listening preferences.

We use a computational model that learns acoustic representations of ‘words’ by processing real spoken utterances that contain the target word, and which are presented in combination with an unambiguous reference to an object corresponding to a specific target word. To provide focus for the experiments, we used strictly supervised learning; as noted above, it has been shown that the model employed in this paper can also learn successfully with ambiguous and partly incorrect references to the objects mentioned in the input utterances [26]. Simulating the indirect feedback that an infant receives during the first months is a more than daunting task [30], and we thus refrain from making any claims in that respect. Overall, the modelling work in this paper relies on clear, unambiguous feedback, as mentioned in the Introduction. This reliance in rendered realistic by only letting the model learn from a few hundred sentences—input infants receive over a few days and which over several months can conceivably occur with unambiguous referents. In designing the model and the learning procedure we aimed at a maximal degree of cognitive and neurophysiological plausibility. The schematic structure of the model, operating in learning mode, is shown in Fig 1; the model in testing mode is shown in Fig 2. In the following sections, we verbally introduce all depicted components in detail. For a formal description, we refer to the Supporting Information, specifically S2 File.

### Input Representations

#### Acoustic input

The model takes real acoustic signals as input, and to be able to simulate infants’ early language processing skills we need a cognitively plausible simulation of the auditory system to convert the signals to a representation that a computer can use for learning. Following a recent survey [31] we assume that infants’ auditory processing system is very similar to the one of adults and that they perceive acoustic signals in terms of their temporal and spectral properties with essentially the same resolution as found in adults. Therefore, we used a conventional acoustic analysis procedure that captures the most important auditory features of the signals. The continuously changing audio signal is divided into short overlapping slices of 20 ms, shifted in steps of 10 ms, so that we obtain 100 spectral envelopes per second. For the spectrum we use 30 band pass filters, equally spaced on a Mel-frequency scale, which corresponds to the frequency resolution in the human auditory system. The way in which the spectral envelopes change over time is perceptually more important than the shape of the envelope (e.g., [32], p. 735). We added first (speed) and second order (acceleration) differences, the so-called Δ and ΔΔ coefficients, to the representation. The output of the 30 band pass filters is heavily correlated and thus forms a very redundant representation. To avoid unnecessary problems in the learning procedures, we applied a cosine transform to the Mel-spectra that reduces the dimensionality from 30 to 13; importantly, the resulting 13 dimensions are uncorrelated. The resulting representation of the speech signal is very similar to the way in which speech is represented in mobile telephony. It is also the preferred representation in speech technology, where it is known as Mel-Frequency Cepstral Coefficients (MFCC) [33]. For a schematic overview of the transformation from the speech signal into MFCCs, see [34]

The tonotopical representations that are formed in the peripheral auditory system can traced up to the auditory cortex [35, 36], albeit with less detail. It appears that some kind of clustering is used for compressing the information. This makes it possible to represent the detailed spectro-temporal information at the output of the acoustic analysis by the centroids of a relatively small number of clusters. It is reasonable to assume that these clusters are formed on the basis of purely acoustic information and the wiring of the brain cells [37] during the early language acquisition. The procedure that we used for replacing individual spectra by the closest cluster centroid is known as Vector Quantisation (VQ). For practical reasons we learned the cluster centroids from recordings of ten speakers of Dutch, which were made under the same conditions as the recordings of the English speakers used in this study. All mobile telephony based on GSM uses VQ to compress the information about the spectral envelopes. The fact that a GSM handset can be used by speakers of any language proves that the VQ procedure is language-independent. We use 150 clusters for the spectral envelopes and for the speed of change values, and 100 clusters for the acceleration. These numbers allow to capture a great amount of detail and are more than sufficient to describe the speech signal without making assumptions regarding the target language. The clusters are referred to with the term *code book labels*.

#### Converting different length utterances to fixed-length representations

If we compute a spectral envelope for each 10 ms stretch of an acoustic signal, which we then represent by a set of three code book labels (one for the stationary envelope, one for the speed of change and a third one for the acceleration), utterances of different lengths will result in representations containing a different number of elements. For all existing learning procedures, probably including learning by mammals, it would be highly beneficial if it were possible to obtain fixed-length representations. If we assume that an audio signal activates tonotopical representations, and if we further assume that these activations will live for the duration of a complex acoustic event, such as a speech signal between two clear pauses, we can convert variable-length signals into fixed-length representations by recording the activation of the complete set of cell assemblies that correspond to the tonotopical representations, because the cardinality of that set is fixed. In our representation each simulated cell assembly corresponds to the co-occurrence of two code book labels at time distances of 20 ms and 50 ms. By using label co-occurrences, instead of individual labels, we represent the dynamic information that is characteristic for speech sounds; these distances can capture both the relative stability within and the changes between adjacent sounds. Larger distances would introduce too much variability, and shorter time lags would lead to overly redundant representations [38]. We present short sentences, which form independent clauses and are typically separated by pauses. Infants can already detect phrase-boundaries at the age of 6 months [39, 40]. Further, the chunking of the signal into short phrases does not over-tax the attentional system. Since all 150+150+100 code book labels may co-occur with all other labels at 20 and 50 ms intervals, the number of different simulated cell assemblies amounts to (150^2^+150^2^+100^2^)*2 = 110,000. Because a one second duration utterance yields 100 sets of co-occurrences, the resulting representations are extremely sparse. There is mounting evidence that sensory inputs are represented in the brain as sparse vectors in a very high dimensional space [41, 42].

The representation of an utterance in the form of counts of co-occurrences is called a Histogram of Acoustic Co-occurrences (HAC; [43]). It can be argued that HAC representations cannot be adequate, because all information about temporal structure beyond 50 ms is lost. However, a representation of spoken utterances as a “bag of acoustic events” has been shown to contain sufficient detail to detect words [44]. It is interesting to note that for the purpose of information retrieval or automatic question answering surprisingly little information is lost if a text is represented as a “bag of words” [45]. Hence, it seems that the contribution of long-distance structures to the interpretation of utterances is somewhat limited. When modelling the initial stages of language acquisition this is very beneficial, since it allows us to “guess” the meaning of an utterance on the basis of a superficial representation.

The code book labels that form the basis for building the HAC representations have been learned from clean speech. We will use the exact same code book labels to convert speech in noise to HAC representations. This implies that we do not provide any form of stream segregation on the basis of the acoustic detail in the audio signals.

#### Meaning representation

In the present paper we used a supervised learning approach. For that purpose we assign a unique and unambiguous label to each utterance in the acoustic material that we use for learning. All utterances in the simulations are simple sentences, and each sentence contains one of 15 different *keywords*, which the label indicates without providing cues to its position within the sentence. In behavioural terms the model will need to learn that a sentence is for example about a cat, and not about mummy or a telephone. In many constrained communication contexts this is probably sufficient to understand the gist of an utterance. The use of complete sentences, instead of isolated words, is motivated by the observation that infants typically are exposed to multi-word utterances [27]. The use of whole sentences also renders the model’s task during both learning and testing more difficult compared to using words in isolation. In all simulations reported here, the keyword is represented by extending the acoustic HAC vectors with a number of entries equal to the number of acoustic-meaning correspondences that must be learned. Each element of the extension corresponds to a single keyword; if the keyword is present in the sentence this entry is set to one; otherwise this entry is set to zero (see Fig 1). We will indicate the HAC vectors enriched with meaning information as HAC+M vectors.

### Learning

It has been shown that complex visual objects are neurally represented as combinations of *primitives*, such as lines and colours [46]. Recent findings about cortical representation of audio signals [35, 36] strongly suggests that a similar procedure operates in auditory perception, which means that complex auditory stimuli are represented as combinations of acoustic primitives. The primitives must be learned from physical stimuli.

Infants necessarily learn from experience; during language acquisition experiences consist of acoustic signals that are perceived in some cross-modal context. In our model learning amounts to discovering acoustic primitives that can be used to distinguish between sentences that relate to one of 15 different objects. These primitives must somehow be discovered by processing HAC+M vectors. We aim for our model to accomplish this in a cognitively and neurophysiologically plausible manner.

HAC+M vectors are an example of very sparse representations in a very high-dimensional space. For high-dimensional sparse representations there are several methods that can be used for simultaneously learning primitives and the way in which complex phenomena are constructed as a sum of the primitives. In our model we chose Non-Negative Matrix Factorization (NMF) [47] as a computational analogue of a cognitive process that updates and modifies internal memory representations based on the experience with perceived stimuli. NMF was originally developed as a batch learning procedure, i.e., a procedure that must iterate over a large database of learning tokens, a procedure that is not cognitively plausible. Driesen, ten Bosch, and Van hamme [48] developed a version of NMF that can be used for incremental and causal learning. Accordingly, our model encounters each utterance in the database of learning materials exactly once, and the primitives are updated after each input utterance.

The algorithms for NMF learning that are available do not allow to learn how many primitives are necessary to represent the learning data. Instead, the number of primitives, in other words the size of the model’s long-term memory, must be specified in advance. It is our experience that this number is not a very important parameter, as long as it is four to five times as large as the number of acoustic-keyword associations that must be learned. Increasing the number of primitives in the memory beyond that number has only marginal effects on the eventual outcome of a learning process. In the simulations for this paper the model needed to learn associations between acoustic signals and 15 keywords. We settled for a model with 70 primitives, which is close to the lower bound of necessary primitives. Thus, the memory in Fig 1 and Fig 2 contains 70 primitives.

When a learning process starts, the primitives in the memory are initialised with small random positive numbers. Processing the HAC+M vector corresponding to the first utterance from the learning material results in updating all primitives in such a way that their sum comes closer to the representation of that HAC+M vector. This leads to the memory representations taking on the same structure as the input, in the present case the memory can therefore be separated into an acoustic and a meaning-encoding part (see Fig 1 and Fig 2). The update process is repeated for all utterances (each represented in a single HAC+M vector) in the learning material. The amount by which the primitives are updated depends on their contribution to approximating the new learning utterance [47]. To avoid overly strong adaptation to the last learning stimulus, the size of adaptations is limited. However, it is important to say that each utterance in the learning material might affect all primitives, not only those that are most strongly associated to the keyword in the input sentence.

In the simulations in this paper the model learns a representation for each keyword. This is enforced by the fact that all sentences that contain a specific keyword have the exact same M subvector in their HAC+M representations, irrespective of the carrier sentence or the speaker who produced the sentence. This implies that the acoustic representations of a keyword must accommodate all the variation that is present in the learning material, be it due to the phonetic and prosodic context, the position of a word in a sentence, the amount of stress put on the word, and so on. Importantly, in the simulations in this study the acoustic primitives were learned from noise-free speech. This allows us to investigate the impact of the linguistic (multiple carrier sentences for the same keyword), para-linguistic (intonation, stress) and extra-linguistic (speaker identity and gender) variation in the learning material on the resilience of the representations that are being learned against noise in the input signals.

### Matching and Recognition

Infants’ behaviour in experiments is often measured in the form of listening preferences (see Introduction for more detail). When infants listen longer (or shorter) to words that they are assumed to know compared to unknown words, the difference is attributed to perceptual and cognitive processing outcomes. Exactly what drives the overt, measurable behaviour of infants who participate in speech perception studies is unclear [16, 17]. The usual interpretation of listening preferences is that infants *recognise* the known stimulus [4, 7, 8], and might be implied that recognition is equivalent to what we mean if we say that an adult person recognises or understands a spoken utterance.

Recognition is associated with a specific abstract concept that is linked to a concrete instance of the recently observed label, such as hearing the word “dog” and thinking of a type of four-legged animal. Recognition can be facilitated by the presence of a visual target, which guides infants’ expectations. This option, while frequently occurring in life outside the lab, is not available in the context of the experiments that form the basis for this paper. Instead, infants only heard words and looked at an unrelated visual target. In this situation, recognition cannot be observed in the form of looks to a visual referent, but must be inferred. Nonetheless, infants might have specific expectations about which word they will hear, for example because they heard this word before. Such a directed attention to and expectation about the presence of a specific target word is what we define as recognition. Recognition, as defined in the context of the present paper, requires the focus of attention on a specific target word. As such, recognition can be used top-down to combat the impact of noise on word recognition. It is unclear whether infants employ such top down mechanisms, but experimental data indicate that infants can cope slightly better with informational masking than with energetic masking [8], which already affects the earliest stages of sensory processing. This points to at least some role of top-down mechanisms.

Experiments in which infants are familiarised with two words, and then tested with familiar or novel words, do not necessarily warrant an interpretation of the responses in terms reminiscent of adult behaviour. Possibly, and more parsimoniously, observable behaviour in this situation is based on some form of *matching* of acoustic representations that do not have any link to meaning representations [17]. It is thus not necessary to invoke any form of expectation and directed attention when explaining infants’ listening behaviour. Any familiar pattern, whether it was recently heard or not, that is detected in the speech input drives behaviour instead and can yield the patterns of responses typically observed in infant experiments comparing two stimulus types. In this context, the following question becomes very relevant from this point of view: Do the unknown words drive less of a reaction because they are not the target (i.e. recognition) or because they are simply a worse match for any known word compared to the target word (i.e. matching)? Current experimental evidence is compatible with both interpretations of infant looking behaviour, namely that the driving mechanism is recognition of specific words or matching stimuli to any known acoustic pattern.

To be able to simulate the outcome measures of infant experiments our model must simulate the equivalent of listening preference. In test mode, depicted in Fig 2, the model does not learn from input and only ‘hears’ the acoustic signal of an utterance (represented as a HAC vector) without the additional meaning (+M vector) information. The NMF algorithm described in the previous section is used to find the weights of the acoustic parts of the 70 primitives in the memory that optimally reconstruct the HAC vector of the test utterance. The same weights are then applied to the meaning part of the 70 primitives in the memory. This results in *activations* for all 15 keywords that are being learned. These activations are a measure of the likelihood that the test utterance contains the corresponding keyword. If the model has successfully learned the associations between the acoustic representations and the keywords, the activation of the presented keyword in the sentence will be larger than the activations of competing words. The acoustic part of the primitives will be able to approximate the HAC representation of any audio signal to some extent. Thus, utterances that do not contain one of the 15 keywords will still result in activations for the keywords. As long as there is no substantial acoustic similarity between unknown words and any of the 15 keywords, it may be expected that all keywords receive a small random activation, and that no one keyword will stand out. We assume that activations and listening time are congruent, so that activations can be considered as (scaled) listening times. The assumption that activation is a scaled representation of listening time ignores all other factors, such as waning attention, that may affect the observable behaviour [17]. Including the systematic and random effects of these factors would increase the variance of the listening time measures, but the overall trends in the results will remain the same.

In our simulations we have implemented both the matching and the recognition interpretations of the perceptual and cognitive processes that are assumed to drive observable behaviour. For both interpretations we derive a measure of listening preference for known words versus foils from the activations of the primitives in the model’s memory. In the *matching* interpretation listening preference is based on the activation of any keyword that receives the highest activation after processing a test utterance. If the test utterance did not contain one of the 15 keywords, the one with the highest activation cannot be ‘correct’. If the test utterance did contain one of the keyword, the identity of that keyword is ignored in the computation o the listening time, which in all cases is based on the activation of the ‘winning’ keyword. In the *recognition* interpretation the listening time is based on the activation of the keyword. If the test utterance contains a foil, rather than a keyword, listening time is still based on the activation of the “impersonated” keyword, since this keyword is expected to occur. It should be noted that the “listening times” cannot be linked to absolute times (which can vary greatly between experiments due to yet unknown factors [16, 49]), hence the relative assessment of comparing target words to foils, akin to many infant studies.

### Design of the Simulation Experiments

#### Speech material

We first present the speech material that provided all auditory input for the model in the present simulations. We used a dedicated speech corpus for the simulations in this paper, which was recorded as part of the ACORNS (ACquisition Of Recognition and communicatioN Skills) project, labelled “Year 2”. The corpus is available upon request at The Language Archive of the Max Planck Institute for Psycholinguistics, via the permanent handle http://hdl.handle.net/1839/00-0000-0000-001A-D60B-1@view.

The recordings were made in a virtually noise-free environment and the speakers were asked to speak as if talking to a young infant. The corpus consists of short English sentences that contain specific *keywords* embedded at initial or final position in various carrier sentences (e.g., “This is a nice ….”, “Where is the happy …?”). Each sentence contains one keyword. We selected 15 words (animal, apple, banana, baby, bird, bottle, car, cat, cookie, daddy, dog, mummy, telephone, toy, truck) as keywords for the simulations. The words were chosen because the data from the MacArthur Communicative Development Inventories (CDI; [50]) suggest that infants growing up in English-speaking countries are familiar with these objects and the corresponding words already in the first year of their life. The corpus contains ten speakers (half of which are female), labelled Speaker 01-10 in the corpus. Four of the ten speakers (two female) produced a number of utterances that was large enough to provide a sufficient amount of speech for learning and testing. One female speaker (Speaker 02 in the corpus) was selected as the ‘primary caregiver’, the speaker from which the model receives all, or most, learning utterances. The other female speaker was used as an unknown test speaker (Speaker 04 in the corpus). Of the remaining eight speakers three males and three females were selected to provide additional speech material in some of the experimental conditions. In addition to the words that were used for learning, we selected the same carrier sentences with unknown words for testing. The unknown words will be indicated by the term *foils*.

In this paper we used multi-talker babble noise from the NOISE-ROM-0, produced in the FP4 ESPRIT Project No. 2589-SAM [51]. We produced test stimuli with SNRs (signal-to-noise ratios) of 10 dB and 5 dB, similar to the SNR values used in Newman’s experiments [4, 7, 8]. The noisy test stimuli were produced by adding the babble noise to the clean speech recordings. The SNR was determined by computing the average Root-Mean-Square power of each of the sentences in the test material, scaling the amplitude of a noise signal of the same duration as the speech signal such that its average Root-Mean-Square power was 10 or 5 dB lower than the speech power, and then adding the two signals. Short-time power variations in the speech signals are much larger than the short-time variation in the babble noise. Therefore, the resulting local SNR in the louder intervals in the speech signals will be larger than the average value, while the softer intervals will have a lower local SNR.

#### Simulating learning before the lab visit

In the experiments conducted by Newman and her colleagues [4, 7, 8] infants were tested to see if they could detect their name when it was spoken repeatedly in the presence of a competing sound. The experiments aimed to investigate the robustness representations that infants formed ‘in the wild’ before they came to the laboratory; the own name is one of the first words that infants seem to detect in noise-free speech in the laboratory [3]. This ability is evidenced by longer listening times to the infant’s name compared to foils.

In the simulations we want to investigate the robustness of word representations that are the result of ecologically plausible learning conditions. Ideally, one would want to simulate learning in noisy environments. However, in the Introduction we have already alluded to the fact that ‘noise’ is a very complex issue: it can be a single competing speaker, many persons speaking at the same time, stationary or non-stationary non-speech noise, covering frequency bands that do or do not overlap with the frequency band that is relevant for speech. Last but not least, the signal-to-noise ratio must be controlled. To make simulation experiments feasible and in the interest of clarity, we decided to use only clean speech during learning and only one noise type during test.

It has been suggested that the representations that infants form of words depend on the number of time they hear a word, and the number of speakers who produce it [52]. An additional factor comes into play when considering that natural language forms infants’ input, namely the frequency with which a word is heard relative to the frequency with which other words are encountered can vary greatly. We first created a *baseline* learning corpus that contains 30 sentences for each of the 15 keywords. The learning material is ordered in blocks of 15 sentences; each block contains one token of each of the keywords. We investigate the effect of the relative frequency of the target word increasing the number of times this word is heard to be twice as large as each of the remaining words. For that purpose we extend the baseline learning corpus with 30 additional utterances that contain the target keyword. The extended corpus consists of 30 blocks of 16 sentences, all spoken by the same female speaker. The effect of hearing the target word spoken is assessed by multiple speakers by extending the baseline corpus with 30 different sentences containing the target word, but now spoken by six additional speakers (speaker 05–10 in the corpus), three female and three male. Each of these six speakers contributed five sentences. The multi-speaker extended learning corpus also consisted of 30 blocks of 16 sentences, one for 14 words, two for one word. The learning conditions are summarised in Table 1.

**Table 1:** Overview of learning conditions.

Experiment	Word Token	# Total	Speakers
1. Baseline	21 to 30	450	1: Primary Caregiver
2. Increased Frequency	42 to 60	480	1: Primary Caregiver
3. Variability from Multiple Voices	21 to 30 + 21 to 30	480	7: Primary Caregiver + 6 Speakers

The first column denotes the experiment, the second the number of word tokens the learner heard at the point of testing, the third column shows the overall number of utterances the learner maximally heard for all *keywords*, the fourth shows the number of speakers observed during learning.

We selected three of the 15 keywords to be treated as target keyword, namely cat, mummy and banana, i.e., a one-, a two- and a three-syllable word. The choice of these three words was random; the ACORNS corpus contains several other one-, two- and three-syllable words. The phonetic and acoustic make-up of the words selected to serve as target word might have an impact on the final results. However, we decided to leave an in-depth investigation of such effects for future research, but we will address the impact of the length and phonetic make-up of the words by comparing the results for cat, mummy and banana.

#### The simulated test

In the assessment of the modelled infant learner three additional factors play a role, namely the *noise level* (no noise, SNR 10, and 5 dB; following infant research summarised above, the *test speaker* (known or unknown), and whether we base our computations of listening times on the assumption that *matching* acoustic patterns is sufficient or that *recognition* of a specific word and its meaning is necessary. The test with clean speech will allow us to determine the performance of the model in fully matched conditions (clean speech in learning and in test). The two different procedures for simulating listening time, based on the *matching* and *recognition* interpretations, are explained in depth above.

For each of the three target words we created two test corpora, each consisting of 80 sentences, 20 containing the respective target word, and three sets of 20 sentences containing one of three matched foils [4, 7]. We selected foils from the part of the ACORNS corpus that was not used for learning. The word cat was matched with the words ball, cow and red; mummy was matched with the words woman, robin and airplane; banana was matched with the words edible, robin and airplane. Obviously, the matches for banana are rather poor in terms of number of syllables and stress pattern, but better matches were not available in the ACORNS corpus. The robin and airplane sentences used in the test with banana were the same as the sentences used in the tests with mummy. One set of test corpora contained sentences spoken by the female speaker who also produced the baseline learning material. Using these test corpora corresponds to a situation in which infants listen to words spoken by their primary caregiver. The other set of test corpora contained speech produced by another female speaker (speaker 04 in the ACORNS corpus) who was not included in the extended multi-speaker learning material. Using these corpora simulates the situation in which infants hear speech produced by an unknown speaker (the test situation in many experimental studies).

During testing, the model listens to the 20 containing the target word and to the three sets of 20 sentences that contain one of the matched foils. For example, when the representation of banana is tested, the model hears the 20 test sentences that contain the word banana, 20 sentences with the foil edible, and so forth. For each of the three target words a single listening preference was computed. For that purpose we summed the normalised activations for the 20 test sentences containing banana and for each of the three sets of 20 foils. Normalisation diminishes the variation between the test sentences, by scaling all activations such that they sum to one for each sentence. The between-sentence variation appeared to be small. Omitting normalisation does not change the results. For the sake of brevity we do not include presence or absence of normalisation as an additional factor in the design of the experiments. Then, the average activation for the utterances with foils is subtracted from the average activations of the utterances with banana. The outcome and thus our main dependent measure is a simulated listening preference, denoting whether or not known words and foils elicited differentiated responses in the model. This model assessment approach can be likened to infant procedures, which also frequently rely on the discrimination of two sets of stimuli (for a similar approach, see [53]). The scale of the simulated listening preference is dependent on the normalisation factor chosen. We thus present preferences, obtaining by subtracting the response to foils from those to target words, if they are above zero we conclude that a systematically different response was elicited by each type of test stimulus.

## Results

In the simulation experiments four fixed factors are relevant: test speaker (known or unknown), past experience (baseline, increased frequency or variable voices, noise level (noise-free, 10 dB SNR, 5 dB SNR), and how preferences were computed (matching or recognition). We also included the specific target word and the sampling point as factors to assess their impact.. While it would have been possible to analyse the results of the simulations with a single linear model, it is more insight-lending and useful to present the results from four simpler statistical models obtained using different parts of the data, since we predicted strong overall outcome differences that would overshadow any further results. We thus built separate models for the two *matching* and *recognition* interpretation in computing listening preference. For both matching and recognition we built models for the known and for the unknown test speaker, since this change in test speaker was expected to lead to an overall lower performance [54]. The models are summarised in Table A to Table D in S1 File. From these Tables it can be seen that the keyword (cat, mummy, banana) makes a significant difference, that the conditions during learning (frequency and the presence of between-speaker variability) interact with the test condition (noise level). Here, we confine ourselves to a verbal and visual presentation of the results to draw attention to the overall patterns found in our modelling outcomes.

It appeared that the factor *sampling point*, the point at which the model’s performance was probed with test items, was almost never significant. The one exception is the experiments with the same speaker during learning and testing when using the recognition-based assessment. Closer inspection of the estimate and the standard deviation reveal that the effect, while statistically significant and thus implying a systematic increase, is very small. In addition, the same underlying model assessed based on matching did not yield such an outcome. Therefore, we will not discuss this factor. All visual presentations are based on the listening preferences averaged over the ten measurement points.

### Known Test Speaker

**Fig 3:**
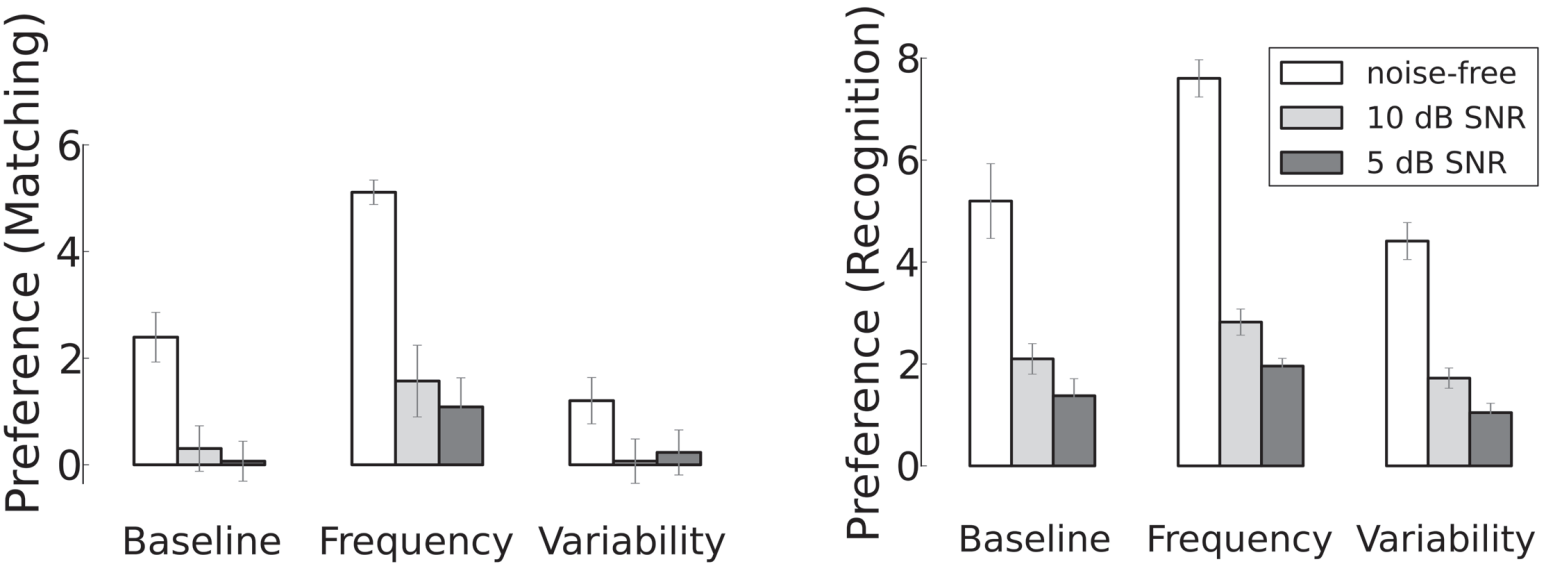
Simulated listening preferences for experiments where the test speaker is known. The two different assessment criteria, general matching-based preference of target word over foils and word recognition-based preference are depicted separately. In all panels and for each experiment the left bar depicts listening preferences without added noise, the middle bar corresponds to 10 dB SNR and the right bar to 5 dB SNR.

We first present the results for the known speaker which are summarised in Fig 3. The left hand panel shows the results for listening preferences based on matching, where the best acoustic match stored in memory was considered; the right hand panel shows the same results for recognition, using only the activations of the intended target word. Both panels contain three sets of listening preference measures, from left to right: the baseline condition, the increased frequency condition, where the target word occurred twice as much as in the baseline, and the multiple speakers condition, in which six new speakers provided the additional tokens of the target word. Each set of bars contains the results for tests in (from left to right) clean speech, 10 dB and 5 dB SNR. In both noise conditions, multi-talker noise from a busy cafeteria was added to the same test material that was used in the clean speech condition. The three bars represent the listening preference averaged over the three words, the whiskers indicate the standard deviation across words and measurements. The height of each bar represents the listening preference, representing the differential responses to target words and foils. The exact values are difficult to link to infant data due to the great variance in the latter [16, 49], we thus mainly focus on whether or not preferences are above zero. For the corresponding numeric values, broken down by individual words, see Table E and Table F in S1 File. While overall the patterns in the left hand and right hand panels are similar, the simulated listening preferences are much larger when they are based on recognition in comparison to matching. The outcomes of the linear models can be found in Table A, Table B, Table C, and Table D in S1 File.

In the baseline condition, where all words occur equally often in the learning material, based on matching there is only a clear listening preference in clean speech. In 10 dB SNR there is still a small listening preference, especially for the words ‘mummy’ and ‘banana’ (see Table E and Table F in S1 File); in 5 dB SNR this only holds for ‘banana’. In the increased frequency condition we see substantially larger listening preferences compared to the baseline and these preferences remain above zero even in 5 dB SNR. In the multiple speaker condition we see a decrease of the listening preference in comparison to the baseline. In 10 dB and 5 dB SNR only the word ‘banana’ shows a preference above zero based on matching in the multiple speaker condition, similar to the baseline condition.

When listening preferences are computed based on recognition, preferences are overall larger compared to the matching condition; but the relative differences between learning and test conditions are smaller than what we have seen in the results based on matching. Preferences always decrease with decreasing SNR (ie. with increasing noise), but they stay above zero in all cases.

The finding that the listening preferences are always larger based on recognition compared to matching is due to the differences in how both were derived from the model’s internal activations upon hearing the same test material (see Fig 2). When computing the recognition listening preferences we only look at the activation for one specific target word. Chances that this activation value is large when the test sentence contains that word, and that the activation value is small(er) when that word is not contained in the test sentence are high. This situation is different for matching. In this case, any word can obtain a large activation value, irrespective of the contents of the test sentence. It may happen that a test sentence with an unknown keyword causes a larger activation of an arbitrary word than a test sentence that contains one of the three singled-out words.

Introducing six additional speakers in the learning material negatively affects the listening preference values when testing with a known speaker. This is due to the presence of data in the learning material (and consequently also in the internal representations used for matching) that is at best irrelevant and possibly even harmful to the task at hand. This holds for matching and recognition, which both show an overall decrease in preference.

Adding noise had the to be expected detrimental effect on listening preferences. The added noise affects the HAC representations of the test sentences in ways that are not straightforward to predict, but that must decrease the correspondence with the representations that were based on clean speech. The impact of the added noise is stronger in 5 dB than in 10 dB SNR, due to the greater alteration of the test material.

The (semi-randomly chosen) target words themselves turned out to have an effect, where ‘cat’ performed worse than ‘banana’. The outstanding performance of ‘banana’ may be related to word length, meaning that it corresponds to a relatively large number of entries in the HAC vector. However, it should also be noted that the foils for ‘banana’ were not very close matches. The weak performance of ‘cat’ may be due to the short duration of that word, in combination with possible overlap in acoustic features between ‘cat’ and other words in the carrier sentences. Future work will have to explore this issue in an in-depth investigation on the role of specific words.

### Unknown Test Speaker

**Fig 4:**
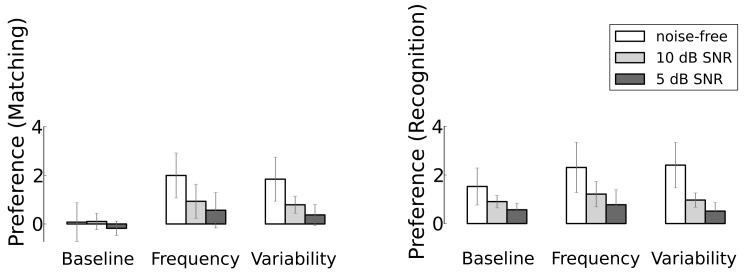
Simulated listening preferences for experiments where the test speaker is unknown. The two different assessment criteria, general matching-based preference of target word over foils and word recognition-based preference, are depicted separately. In all panels and for each experiment the left bar depicts listening preferences without added noise, the middle bar corresponds to 10 dB SNR and the right bar to 5 dB SNR.

The results of the simulations with the known speaker during testing might overestimate the robustness of the representations, because in actual practice infants usually listen to speech produced by an unknown speaker. To investigate the robustness of the representations when testing with speech of an unknown speaker, we computed listening preferences with the exact same material as used in the experiments described above, but spoken by a different female speaker. The results of these simulations are summarised in Fig 4; for the details of what is displayed in the figure, see the explanation of Fig 3.; the corresponding numerical values and linear models can be found in S1 File (Tables A to D).

The listening preferences with the unknown speaker during test are overall smaller than with the known speaker. This result was to be expected based on previous experiments [54]. The internal representations are the same as in the tests with the known speaker since they stem from the same model and they were exposed to the same learning material. The only difference is that now the test material is produced by an unknown female speaker. All changes in listening preferences must therefore be due to the fact that the test material now matches less well with the representations that were learned from the speech of another speaker (or, in the condition with multiple voices: of other speakers). Although the difference is much smaller than for the known test speaker, the listening preferences obtained with recognition are larger than with matching. When the results are based on matching (left panel) the baseline condition, in which all words were presented equally often, did not show an overall listening preference for the known words over the foils. Only ‘banana’ seems to generate a slightly higher preference in noise-free test sentences and at a noise-level of 10 dB SNR. The effect of the added noise is small for this word (if it is at all present).

In the increased frequency and multiple speaker conditions the detrimental effect of the added noise is evident: listening preferences in 10 dB SNR are lower than with clean speech, and in 5 dB SNR the preferences are even lower. However, it is also clear that the effect of the noise is much smaller than with the known test speaker. This finding can be interpreted in two ways: either the effect of noise on the listening preference for the known speaker is exaggerated, because every manipulation of the speech of the known speaker should result in a worse match with her speech in the test. Or the finding shows that the representations of the three singled-out words are fairly robust since they are adequate for an unknown speaker, and only weakly affected by the added noise.

The effect of adding additional learning tokens from the primary caregiver is the same as when adding additional tokens from six other speakers. Both conditions increase the relative amount of learning material for the singled-out words. Still, the amount of variation contributed by the additional tokens of the primary caregiver is smaller than the variation added by the six other speakers. The additional variation contributed by multiple speakers does not increase the listening preference with the unknown speaker. This suggests that adding variation to the learning material is not very effective if that variation does not correspond to the idiosyncratic properties of the speech of the test speaker.

The most striking difference between matching and recognition with the unknown speaker is in the baseline condition. Apparently, the representations of the target words, and by implication the representations of all words, are already sufficiently powerful—and sufficiently robust against a speaker change—after processing 21 to 30 learning tokens per word to distinguish sentences that contain a target word from sentences that contain foils.

## General Discussion

In the present paper we employed a computational model to investigate infants’ early speech processing abilities in adverse conditions. Our simulations addressed the question which processes and knowledge are necessary to explain infant behaviour observed in experimental studies. To this end, we simulated word detection in two key noise conditions frequently employed in infant studies, namely 10 and 5 dB SNR. The overall goal was to replicate the behavioural pattern that infants succeed in most word detection tasks with 10 dB SNR background noise and show greater difficulty at 5 dB SNR [1, 4]. Our results show that a model with general-purpose speech processing abilities which does not implement any form of bottom-up stream segregation suffices to explain infants’ abilities as measured in a number of experimental studies.

Three factors that we addressed in this paper, next to building an overall credible model of infants’ early linguistic abilities, strongly influenced the simulated behaviour and we discuss each in turn. First, we either used the same speaker during learning and in the test phase or a completely unknown speaker. The latter case is the standard situation in infant studies. As expected, based on both previous infant studies [1, 15] and preliminary modelling work [54], overall performance was much lower when the speaker had not been part of the learning material. Nonetheless, the model (partly) succeeded in recognising words in noise even in the typical test situation with an unknown speaker, lending further credibility to the present modelling approach.

Second, we assessed the role of previous experience, manipulating the amount of exposure and whether or not multiple speakers had spoken the target word in the learning phase. Variability between speakers has repeatedly been suggested to aid the formation of more mature, and presumably more noise-robust representations [4, 14]. Overall, more experience improved noise-robustness. However, the impact of previous experience interacted with the first factor, namely whether or not the test speaker was known. When testing with a known speaker, increasing the number of sentences spoken by the same speaker proved most beneficial and the model successfully detected words across noise conditions. Adding more speakers to the learning material, in contrast, yielded no improvement when the same speaker provided most of the learning and all of the test sentences.

Third, we compared two possible processes that might underlie infants reactions to presumably known and unknown words. Either infants simply compare acoustic patterns in their memory to the experimental stimuli, termed matching, or they focus on a specific word and its meaning, they recognise a word (or its absence). Matching, as defined within this paper, is sufficient to simulate the results of infant studies, and thus might account for the processing strategy employed by infants in the second half of their first year. Recognition proved to be more noise-robust, which is due to the attentional focus on a specific word and its meaning. The extent of the simulated noise-robustness exceeded the abilities of 6- to 8-month-olds and is more in line with abilities infants only display around their first birthday [4]. Multiple other changes might account for infants’ improved ability to deal with background noise, but considering the parallel maturation of the attentional system it seems plausible to attribute this to a change from acoustic matching to targeted recognition. Notably, infants’ abilities not only improve in the context of ambient noise; word recognition in the presence of a visual target also advances drastically, as shown for example by Bergelson and Swingley [13]. Maturing attentional skills and a more robust link of acoustic patterns and their meaning can also account for the type of recognition measured when infants have to focus on the correct image upon hearing its label.

Our simulations provide an interpretative framework for the small number of behavioural experiments that investigated the resilience of infants’ speech processing to additive noise, e.g., [1, 4]. The first papers on the impact of noise on infant speech processing attributed substantial importance to auditory stream segregation. It should be noted that more recent work does not invoke stream segregation as an explanatory mechanism [8]. In the Introduction we explained why it is unlikely that infants could be able to deploy stream segregation in an experimental setting in which the most powerful tools that adults use for stream segregation, directional hearing and observing lip movements, were not available. To avoid making unwarranted assumptions about infants’ abilities, we refrained from implementing stream segregation in our model. Future work can address directional hearing; behavioural experiments might for example ploy several loudspeakers as sources for both the target speech signal and the distracting noise. Admittedly, additional acoustic measurements are necessary to obtain a specific SNR at the position of the infant’s head, but at the very least such an experimental set up could answer the question to what extent infants can use directional hearing when hearing speech in noise. If infants use binaural hearing to separate signals, they should outperform participants tested in the manner simulated here, namely with a signal that has been merged a priori and is presented from one physical source.

In the Introduction we pointed out the tension between behavioural experiments, in which infants’ skills of the day are measured irrespective of how and when they have been acquired on the one hand and simulation experiments, in which the emphasis is on the learning process on the other hand. Arguably, simulation experiments are closer to theories about language acquisition [29] than most behavioural experiments, since they make all assumptions explicit and take into account which abilities and experiences led to an observed behaviour in a laboratory test. Our simulations add to a comprehensive theory of language acquisition by questioning whether the conclusions drawn from behavioural experiments ignore alternative processes that could have yielded the same observable behaviours. As such, the present work has implications when interpreting infant data beyond the scope of speech perception in noise.

The way in which audio signals are represented in our model (as sparse vectors in a very high-dimensional space) and the way in which associations are learned between acoustic and meaning representations (by means of sparse coding, implemented as Non-negative Matrix Factorisation) are very different from previous models of language acquisition. However, our approach is strongly supported by recent findings in neurobiology and neurocognition [41, 42, 46]. The stimuli for learning and for testing consisted of short sentences. Neither during learning, nor during processing in a test an attempt was made to segment words from the sentences. If words were presented in isolation, either during learning or in test, the model’s task would have been considerably easier, due to the fact that the whole acoustic signal is the target. In this case, performance would have been substantially improved. However, the overall patterns of results would remain the same.

The results of the simulations showed that it is possible to distinguish between test sentences that contain a known word and test sentences that contain unknown words (foils) in most test conditions without segmenting the sentences into words. This corroborates one of the basic tenets of PRIMIR. An important implication of this finding, that to our knowledge is seldom discussed in the literature on language acquisition, is that infants can react appropriately to spoken utterances well before they are able to perform advanced linguistic operations on the speech signal. This provides a powerful scaffolding structure to bootstrap into language leaving abstract, symbolic representations to be acquired later, instead of being a necessary precursor to detecting and reacting to known words. Equally importantly, the model could distinguish between known and unknown words in noisy speech without applying any form of stream segregation, which suggests that infants do not need to rely on sophisticated segregation capabilities in early language acquisition and still can make use of the information present even in a noisy speech signal.

With the recognition interpretation of the underlying cognitive process the listening preferences that we found in tests with 10 dB and 5 dB SNR seem to exceed the abilities shown by young infants in laboratory experiments [1, 4]. From this we concluded that at least part of the behaviour observed in these experiments should be attributed to acoustic matching, rather than to recognition of the meaning of test stimuli, even if the stimuli consist of repeated productions of the infant’s name. To disentangle whether infants perform a form of specific word recognition or general acoustic matching, we suggest to manipulate not the target word but the foils. When infants have to process a target word, such as their own name or a word that has previously been familiarised, the foils can be manipulated in their similarity to other words which the infant is assumed to know, such as “mommy”. It would further be interesting to investigate whether infants who are not able to connect putatively known words to the corresponding picture would still show a listening preference for those words relative to acoustically dissimilar words. Such studies can further illuminate which representations underlie the observable behaviour in infant experiments.

Our simulations showed that hearing more tokens of a word helps in distinguishing that word from foils, but the effect differed both depending on whether one or multiple speakers provided the additional input and whether or not the test speaker was known. This suggests that while variation in the learning material is relevant, the effect of the variation on some behavioural measure depends to a large extent on characteristics of the test stimuli. The larger the mismatch is between the distributions in the learning material and the features of the test material, the lower the performance will become. In interpreting the results of previous experiments and simulations of the contribution of variation in the learning material (e.g., [19, 21]) and in the design of future experiments, care must be taken to assess the extent to which the test procedure is hampered or enhanced by the type and amount of variation infants experienced before the lab visit.

This first attempt to simulate speech processing in noisy conditions has many limitations that need to be addressed in future research. Although the representation of speech in the form of Mel-Frequency spectra allows to distinguish female and male speakers (mainly based in the presence or absence of energy in the filters with the lowest centre frequencies), a more explicit representation of voice pitch might help in separating competing speakers. However, it is quite possible that this mechanism for stream segregation only becomes effective if other mechanisms, especially those that require some form of understanding and prediction, have become available. Future simulations should also take into account potential differences between infants in the way in which they manifest the results of perceptual and cognitive processing. Specifically, we limited learning to noise-free speech; future work should investigate the impact of noise in the learning material, since infants are exposed to noisy language input in their daily lives [1].

In the present work only three words from a fixed lexicon of 15 words were used. For clarity, we limit ourselves to the presented results, and instead of only discussing averages point to observed differences between words, namely that the shortest word, ‘cat’ led to substantially worse performance than the longest word, ‘banana’ (see Table E and Table F in S1 File). While we observed an effect of the specific words, a detailed investigation requires the use of multiple words, different lexicon sizes and word combinations, and ideally also the use of multiple languages to avoid a bias towards one linguistic system. We expect that the overall results presented here can be replicated, but it is premature to speculate about the origin of the differences between the words.

HAC+M vectors are reminiscent of distributed representations: The counts in a vector can be interpreted as connection strength between cell assemblies in the brain. The fact that our HAC+M vectors are composed of two sub-vectors can be interpreted as representing connections between regions in the brain (or planes in PRIMIR). In this light, it is interesting that NMF learning can be linked to learning in multi-layer perceptrons [55]. Having said this, it must be added that the representations that are formed by NMF learning cannot be equated to nodes in a neural network. For this reason it is premature to speculate about possible relations between the distributed representations in our model and the distributed cohort model proposed by Gaskell and Marslen-Wilson [56] that uses a recurrent neural network to learn associations between phonetic features, phonemes and words. For the time being our model is only explicit about representations on the General Perceptual Plane in PRIMIR. The representations on higher-level planes, which provide the supervision in the learning process, cannot make any claim towards specificity or cognitive and neurophysiological plausibility. Extending the model and the learning procedures such that the language-specific representations become explicit and plausible is probably the biggest challenge to be met in future research.

## Supporting Information

S1 File Tables displaying the numerical results of a linear models with two each for the known test speaker condition (*matching*: Table A; *recognition*: Table B) and for the unknown test speaker condition (*matching*: Table C; *recognition*: Table D) The final two tables display simulated listening preferences for both *matching* (Table E) and *recognition* (Table F). Table A, Results of a linear model based on matching when the speaker is known. Results for the linear model based on *matching* when the speaker is known. Significance indicators (uncorrected): * *p* <.05,** *p* <.01,*** *p* <.001. Table B, Results of a linear model based on recognition when the speaker is known. Results for the linear model based on *recognition* when the speaker is known. Significance indicators (uncorrected): * *p* <.05,** *p* <.01,*** *p* <.001. Table C, Results of a linear model based on matching when the speaker is unknown. Results for the linear model based on *matching* when the speaker is unknown. Significance indicators (uncorrected): * *p* <.05,** *p* <.01,*** *p* <.001. Table D, Results of a linear model based on recognition when the speaker is unknown. Results for the linear model based on *recognition* when the speaker is unknown. Significance indicators (uncorrected): * *p* <.05,** *p* <.01,*** *p* <.001. Table E, Simulated listening preferences based on matching. Simulated listening preferences based on *matching* (mean and standard deviation) for all conditions. Listening preferences that are significantly above 0 are indicated (based on an uncorrected one-sided *t*-Test): * *p* <.05,** *p* <.01,*** *p* <.001. Table F, Simulated listening preferences based on recognition. Simulated listening preferences based on *recognition* (mean and standard deviation) for all conditions. Listening preferences that are significantly above 0 are indicated (based on an uncorrected one-sided *t*-Test): * *p* <.05,** *p* <.01,*** *p* <.001.
(PDF)

S2 File A formal description of all modelling work described in this paper and implemented in the accompanying scripts. (PDF)
