# Supplementary material for: Modelling the Noise-Robustness of Infants’ Word Representations: The Impact of Previous Experience
Source: PLoS One. 2015 Jul 28;10(7):e0132245. doi: 10.1371/journal.pone.0132245 (PMC4517927; doi:10.1371/journal.pone.0132245)
Supplement: S1 File — Table A, Results of a linear model based on matching when the speaker is known. Results for the linear model based on matching when the speaker is known. Significance indicators (uncorrected): * p <.05,** p <.01,*** p <.001. Table B, Results of a linear model based on recognition when the speaker is known. Results for the linear model based on recognition when the speaker is known. Significance indicators (uncorrected): * p <.05,** p <.01,*** p <.001. Table C, Results of a linear model based on matching when the speaker is unknown. Results for the linear model based on matching when the speaker is unknown. Significance indicators (uncorrected): * p <.05,** p <.01,*** p <.001. Table D, Results of a linear model based on recognition when the speaker is unknown. Results for the linear model based on recognition when the speaker is unknown. Significance indicators (uncorrected): * p <.05,** p <.01,*** p <.001. Table E, Simulated listening preferences based on matching. Simulated listening preferences based on matching (mean and standard deviation) for all conditions. Listening preferences that are significantly above 0 are indicated (based on an uncorrected one-sided t-Test): * p <.05,** p <.01,*** p <.001. Table F, Simulated listening preferences based on recognition. Simulated listening preferences based on recognition (mean and standard deviation) for all conditions. Listening preferences that are significantly above 0 are indicated (based on an uncorrected one-sided t-Test): * p <.05,** p <.01,*** p <.001. (PDF) [file pone.0132245.s001.pdf]

S1 File

Table A

Table 1. Supporting Table A: Results of a linear model based on matching when the speaker is known.

**Residuals:**					
Min	1Q	Median	3Q	Max	
-1.19	-0.21	-0.02	0.20	1.48	
**Coefficients:**					
	**Estimate**	**Std.Error**	**t value**	**Pr(>t)**	
(Intercept)	0.62	0.08	7.16	<.001	***
Experiment: Increased Frequency	1.30	0.09	13.47	<.001	***
Experiment: Multiple Voices	-0.26	0.09	-2.69	.007	**
Noise Level: 5 dB SNR noise	-0.23	0.09	-2.44	.015	*
Noise Level: noise-free	2.08	0.09	21.53	<.001	***
Cat	-0.70	0.05	-12.54	<.001	***
Mummy	-0.49	0.05	-8.90	<.001	***
Sample point	0.01	0.007	1.77	.07	
Increased Frequency:5 dB SNR noise	-0.23	0.13	-1.68	.09	
Multiple Voices:5 dB SNR noise	0.41	0.13	3.05	.002	**
Increased Frequency:noise-free	1.41	0.13	10.30	<.001	***
Multiple Voices:noise-free	-0.91	0.13	-6.64	<.001	***

Results for the linear model based on *matching* when the speaker is known. Significance indicators (uncorrected): $^{*}\ p < .05$, $^{**}\ p < .01$, $^{***}\ p < .001$

Table B

Table 2. Supporting Table B: Results of a linear model based on recognition when the speaker is known.

**Residuals:**					
Min	1Q	Median	3Q	Max	
-1.65	-0.39	0.01	0.33	2.15	
**Coefficients:**					
	**Estimate**	**Std.Error**	**t value**	**Pr(>t)**	
(Intercept)	2.21	0.13	16.44	<.001	***
Experiment: Increased Frequency	.00	0.14	6.73	<.001	***
Experiment: Multiple Voices	-2.36	0.14	-15.89	<.001	***
Noise Level: 5 dB SNR noise	-0.84	0.14	-5.64	<.001	***
Noise Level: noise-free	3.00	0.14	20.18	<.001	***
Cat	0.01	0.08	0.19	.84	
Mummy	-0.58	0.08	-6.80	<.001	***
Sample point	0.03	0.01	2.87	.004	**
Increased Frequency:5 dB SNR noise	-0.19	0.21	-0.93	.34	
Multiple Voices:5 dB SNR noise	1.32	0.21	6.30	<.001	***
Increased Frequency:noise-free	1.52	0.21	7.23	<.001	***
Multiple Voices:noise-free	-1.76	0.21	-8.36	<.001	***

Results for the linear model based on *recognition* when the speaker is known.
Significance indicators (uncorrected): * $p < .05$,** $p < .01$,*** $p < .001$

Table C

Table 3. Supporting Table C: Results of a linear model based on matching when the speaker is unknown.

**Residuals:**					
Min	1Q	Median	3Q	Max	
-1.15	-0.25	-0.007	0.21	1.22	
**Coefficients:**					
	**Estimate**	**Std.Error**	**t value**	**Pr(>t)**	
(Intercept)	0.74	0.08	8.45	<.001	***
Experiment: Increased Frequency	0.84	0.09	8.70	<.001	***
Experiment: Multiple Voices	0.68	0.09	7.07	<.001	***
Noise Level: 5 dB SNR noise	-0.28	0.09	-2.90	.004	**
Noise Level: noise-free	-0.02	0.09	-0.28	.77	
Cat	-1.30	0.05	-23.24	<.001	***
Mummy	-0.72	0.05	-12.85	<.001	***
Sample point	0.006	0.007	0.82	.40	
Increased Frequency:5 dB SNR noise	-0.08	0.13	-0.61	.53	
Multiple Voices:5 dB SNR noise	-0.11	0.13	-0.85	.39	
Increased Frequency:noise-free	1.10	0.13	8.04	<.001	***
Multiple Voices:noise-free	1.09	0.13	7.92	<.001	***

Results for the linear model based on *matching* when the speaker is unknown.
Significance indicators (uncorrected): * $p < .05$,** $p < .01$,*** $p < .001$

Table D

Table 4. Supporting Table D: Results of a linear model based on recognition when the speaker is unknown.

**Residuals:**					
Min	1Q	Median	3Q	Max	
-1.09	-0.19	0.01	0.22	1.19	
**Coefficients:**					
	**Estimate**	**Std.Error**	**t value**	**Pr(>t)**	
(Intercept)	1.90	0.09	20.81	<.001	***
Experiment: Increased Frequency	0.51	0.10	5.11	<.001	***
Experiment: Multiple Voices	-0.25	0.10	-2.53	.011	*
Noise Level: 5 dB SNR noise	-0.37	0.10	-3.65	<.001	***
Noise Level: noise-free	0.58	0.10	5.80	<.001	***
Cat	-1.30	0.05	-22.35	<.001	***
Mummy	-1.22	0.05	-20.95	<.001	***
Sample point	0.004	0.008	0.59	.55	
Increased Frequency:5 dB SNR noise	-0.12	0.14	-0.85	.39	
Multiple Voices:5 dB SNR noise	-0.06	0.14	-0.46	.64	
Increased Frequency:noise-free	0.89	0.14	6.22	<.001	***
Multiple Voices:noise-free	0.81	0.14	5.70	<.001	***

Results for the linear model based on *recognition* when the speaker is unknown.
Significance indicators (uncorrected): * $p < .05$,** $p < .01$,*** $p < .001$

Table E

Table 5. Supporting Table E: Simulated listening preferences based on matching.

Experiment	Word	SNR					
		noise-free		10 dB		5 dB	
**Known speaker during testing**							
1. Baseline							
	cat	2.76 (0.53)	***	-0.15 (0.32)		-0.38 (0.09)	
	mummy	2.14 (0.11)	***	0.34 (0.21)	***	0.13 (0.22)	
	banana	2.28 (0.38)	***	0.72 (0.15)	***	0.46 (0.11)	***
2. Increased							
Frequency	cat	5.07 (0.15)	***	1.10 (0.12)	***	0.49 (0.14)	***
	mummy	5.13 (0.19)	***	1.09 (0.25)	***	0.91 (0.29)	***
	banana	5.35 (0.20)	***	2.40 (0.26)	***	1.68 (0.35)	***
3. Multiple							
Voices	cat	0.91 (0.25)	***	-0.24 (0.15)		-0.13 (0.09)	
	mummy	1.83 (0.21)	***	-0.26 (0.25)		-0.07 (0.22)	
	banana	1.15 (0.15)	***	0.60 (0.13)	***	0.77 (0.15)	***
**Unknown speaker during testing**							
4. Baseline							
	cat	-0.71 (0.13)		-0.29 (0.19)		-0.37 (0.10)	
	mummy	0.11 (0.16)		0.17 (0.15)	**	-0.38 (0.09)	
	banana	0.82 (0.84)	*	0.43 (0.14)	***	0.21 (0.15)	**
5. Increased							
Frequency	cat	0.61 (0.52)	**	-0.02 (0.10)		-0.39 (0.08)	
	mummy	1.97 (0.37)	***	0.86 (0.15)	***	0.43 (0.25)	***
	banana	2.80 (1.00)	***	1.67 (0.24)	***	1.39 (0.29)	***
6. Multiple							
Voices	cat	1.41 (0.39)	***	0.29 (0.14)	***	-0.23 (0.10)	
	mummy	0.93 (0.61)	**	0.78 (0.14)	***	0.48 (0.15)	***
	banana	2.97 (0.85)	***	1.04 (0.15)	***	0.68 (0.21)	***

Simulated listening preferences based on *matching* (mean and standard deviation) for all conditions. Listening preferences that are significantly above 0 are indicated (based on an uncorrected one-sided t-Test): * $p < .05$,** $p < .01$,*** $p < .001$

Table F

Table 6. Supporting Table F: Simulated listening preferences based on recognition.

Experiment	Word	SNR noise-free		SNR 10 dB		SNR 5 dB	
**Known speaker during testing**							
1. Baseline							
	cat	5.98 (0.66)	***	2.16 (0.27)	***	1.33 (0.21)	***
	mummy	4.74 (0.17)	***	2.31 (0.12)	***	1.75 (0.12)	***
	banana	4.87 (0.47)	***	1.82 (0.23)	***	1.05 (0.17)	***
2. Increased Frequency	cat	7.90 (0.21)	***	3.03 (0.17)	***	1.83 (0.15)	***
	mummy	7.29 (0.28)	***	2.48 (0.23)	***	1.95 (0.27)	***
	banana	7.62 (0.20)	***	2.70 (0.29)	***	2.70 (0.29)	***
3. Multiple Voices	cat	4.40 (0.53)	***	1.51 (0.21)	***	0.82 (0.17)	***
	mummy	4.40 (0.28)	***	1.65 (0.24)	***	1.01 (0.23)	***
	banana	4.31 (0.22)	***	1.81 (0.22)	***	1.07 (0.27)	***
**Unknown speaker during testing**							
4. Baseline							
	cat	0.68 (0.10)	***	0.62 (0.09)	***	0.28 (0.05)	***
	mummy	1.51 (0.27)	***	0.90 (0.11)	***	0.53 (0.08)	***
	banana	2.39 (0.44)	***	1.19 (0.15)	***	0.89 (0.13)	***
5. Increased Frequency	cat	0.76 (0.48)	**	0.49 (0.11)	***	-0.08 (0.07)	
	mummy	2.26 (0.42)	***	1.03 (0.16)	***	0.55 (0.21)	***
	banana	3.28 (0.98)	***	1.78 (0.26)	***	1.46 (0.28)	***
6. Multiple Voices	cat	2.35 (0.43)	***	0.55 (0.14)	***	-0.01 (0.12)	
	mummy	1.06 (0.60)	***	0.93 (0.19)	***	0.67 (0.16)	***
	banana	3.62 (0.74)	***	1.14 (0.23)	***	0.61 (0.28)	***

Simulated listening preferences based on *recognition* (mean and standard deviation) for all conditions. Listening preferences that are significantly above 0 are indicated (based on an uncorrected one-sided t-Test): $^{*}\ p < .05$,$^{**}\ p < .01$,$^{***}\ p < .001$
