# Supplementary material for: Modelling the Noise-Robustness of Infants’ Word Representations: The Impact of Previous Experience
Source: PLoS One. 2015 Jul 28;10(7):e0132245. doi: 10.1371/journal.pone.0132245 (PMC4517927; doi:10.1371/journal.pone.0132245)
Supplement: S2 File — (PDF) [file pone.0132245.s002.pdf]

Input representation

Acoustic encoding

Mel-Frequency Cepstral Coefficients

Mel-Frequency Cepstral Coefficients (MFCCs) are a popular representation of the time-varying spectral characteristics of speech signals in Automatic Speech Recognition. Let $s_t, t = 0, 1, \cdots, T$ be the discrete-time representation of a continuous speech signal $s(t)$. To account for the tendency that the energy in speech signals is concentrated in the lower frequencies, the signal s_t is first differenced so as to yield $\hat{s}_t = s_t - .97 \times s_{t-1}$. From the signal $\hat{s}_t$ overlapping intervals with a duration of 20 ms are extracted by multiplying $\hat{s}_t$ by a Hamming window w_t that is shifted in steps of 10 ms:

$$w(n) = 0.54 - 0.46 \cdot \cos\left(\frac{2 \cdot \pi \cdot n}{K-1}\right), n = 0, 1, \cdots, K$$

An utterance with a duration of, for example, 3 s (= 3000 ms) will result in a sequence of 300 speech frames.

To transform the signal from the time domain into the spectral domain, a Discrete Fourier Transform (DFT) is calculated for each windowed speech frame via

$$|X_f|^2 = |\sum_{n=0}^{N-1} (\hat{s}(n) \cdot w(n)) \cdot e^{-i2\pi \cdot n \cdot f/N}|^2 \tag{1}$$

with N being the number of DFT frequencies (set to 400 in the present paper). The absolute values of the resulting $N/2$ Fourier coefficients are then multiplied by the triangular frequency response of 30 bandpass filters

with center frequencies defined on the technical Mel frequency scale with $m \approx 2595 \cdot \log_{10}\left(1 + \frac{f}{700}\right)$ for frequencies $f > 700$ Hz, and a linear relation between m and f for frequencies < 700 Hz. This arrangement corresponds to the frequency resolution of the human auditory system. The weighted Fourier coefficients are summed to obtain 30 Mel-frequency spectral energy coefficients, of which the 10-log is taken. Finally, the 30 Mel-spectral power values MF_q are converted to 12 MFCCs by means of an Inverse Discrete Cosine Transform:

$$\mathrm{MFFC}_m = \sum_{q=0}^{30} \sqrt{\frac{2}{30}} \cdot \log(\mathrm{MF}_q) \cos\left(\frac{2\pi \cdot (m-1) \cdot (q-1)}{2 \cdot 30}\right) \tag{2}$$

with $m = 1, 2, \cdots, 12$. The log-energy is added as the 13th coefficient. The Δ and $\Delta\Delta$ coefficients are computed from the 13 coefficients as the linear regression over time in a sequence of nine adjacent frames. The result is a 39 dimensional vector, updated every 10 ms.

Vector Quantisation

Each time frame of the signal is represented as a set of 13 static MFCC, 13 Δ, and 13 $\Delta\Delta$ coefficients, i.e., a vector consisting of three sets of 13 real numbers. To limit the number of possible representations Vector Quantisation (VQ) is applied to the three vectors. To this end, three code books of 150, 150, and 100 labels for the MFCC, Δ, and $\Delta\Delta$ coefficients, respectively, were obtained a priori based on conventional k-Means clustering applied to the MFCC analysis of recordings made of ten native speakers of Dutch, who read short sentences in a noise-free environment. After the VQ step, each

speech frame is represented by three VQ labels; one from each of the three code books. Per code book, the label $l_j(a_t)$ for a speech frame a_t corresponds to the index of the code book prototype $p_{i,j}$ that has the smallest Euclidean distance to a_t:

$$l_j(a_t) = \underset{i}{\operatorname{argmin}}(a_t - p_{i,j})^2, \quad j = 1, 2, 3. \tag{3}$$

The VQ labels are largely language independent: we experimented with sets of labels obtained from different languages, and found no differences.

Histogram of Acoustic Co-ocurrences

As a result of the VQ operation, each utterance is represented as a sequence of triplets of VQ labels. Utterances of unequal duration will result in sequences of triples of VQ labels of unequal length. To obtain a fixed-length representation, the sequence of triples of VQ labels of an utterance is converted into a Histogram of Acoustic Co-occurrences (HAC; (?, ?)). A HAC representation is a (very high dimensional) vector that contains for each pair of VQ labels the number of times that these labels co-occur at a distance of two and at a distance of five frames. Since there are 150 labels for the static MFCCs, 150 labels for the Δ, and 100 labels for the $\Delta\Delta$, there are $2 \times 150^2 + 2 \times 150^2 + 2 \times 100^2$ possible co-occurrences. This results in HAC

vectors of the form

$$V_a = \begin{bmatrix} V_{\mathrm{MFCC}}^{lag=2} \\ \cdot \\ V_{\mathrm{MFCC}}^{lag=5} \\ \cdot \\ V_{\Delta}^{lag=2} \\ \cdot \\ V_{\Delta}^{lag=5} \\ \cdot \\ V_{\Delta\Delta}^{lag=2} \\ \cdot \\ V_{\Delta\Delta}^{lag=5} \end{bmatrix} \qquad (4)$$

A signal of 3 s generates close to 600 counts in the $110,000$-dimensional HAC vector, which amounts to a sparseness of 99.45 % if all these counts fall into different HAC components. It is likely that some of them co-contribute to the same component, resulting in sparseness at $>$ 99.45 %. Therefore, HAC representations of short utterances are extremely sparse.

Meaning encoding

The HAC vectors V_a that represents the acoustic information of an utterance are augmented with a (much shorter) extension V_m which represents the meaning of an utterance. In this paper *the meaning of an utterances* is defined as the presence of a specific *keyword* in that utterance. This information can be encoded in a vector with the length of the number of possible

keywords, with a value of one at the index position of the keyword, and a value of zero at all other index positions:

$$V_m[i] = \begin{cases} 1 & \text{if the utterance contains keyword } i \\ 0 & \text{otherwise} \end{cases} \quad (5)$$

Learning and matching: Non-negative Matrix Factorization

Non-negative Matrix Factorization (NMF; ?, ?) is used for learning associations between the acoustic and meaning representations and or finding the best match between learned representations and unknown input during tests. The general idea, as introduced by ? (?), is as follows: An input matrix $\mathbf{V}$ is of size $m \times n$, with m being the dimension with which perceptual input is encoded (here more than $110,000$, as described in the previous section), and n referring to the number of observations. NMF factorises $\mathbf{V}$ as two much smaller matrices $\mathbf{W}$ and $\mathbf{H}$, of size $m \times r$ and $r \times n$ respectively, with $r \ll m, n$, such that

$$\mathbf{V} \approx \mathbf{W} \times \mathbf{H}. \quad (6)$$

This factorisation expresses each column of $\mathbf{V}$ in terms of a linear combination of limited number of vectors in $\mathbf{W}$, whose representational format is the same as $\mathbf{V}$, but the memory size is limited by the inner dimension r. The matrix $\mathbf{H}$ contains the weights required to represent $\mathbf{V}$ in terms of the contents of $\mathbf{W}$ and can be considered as temporary connections between internal representations.

The cost function that was used in NMF is the Kullback-Leibler (KL) divergence, which governs the approximation described in equation **??**.

$$D_{KL}(\mathbf{WH}\|\mathbf{V}) = \sum_{ij}(\mathbf{V}_{ij} log \frac{\mathbf{V}_{ij}}{(\mathbf{WH})_{ij}} + (\mathbf{WH})_{ij} - \mathbf{V}_{ij}) \tag{7}$$

The NMF operation is implemented by iteratively applying the following steps (in the present work we limited the number of operations to 2):

$$\begin{aligned}
&\mathbf{W}_{ik} \leftarrow \mathbf{W}_{ik} \sum_j \mathbf{H}_{kj} \left(\frac{\mathbf{V}}{\mathbf{WH}}\right)_{ij} \\
&Normalise : \sum_i \mathbf{W}_{ik} = 1 \\
&\mathbf{H}_{kj} \leftarrow \mathbf{H}_{kj} \sum_i \mathbf{W}_{ik} \left(\frac{\mathbf{V}}{\mathbf{WH}}\right)_{ij} \\
&Normalise : \sum_i \mathbf{H}_{ik} = 1
\end{aligned} \tag{8}$$

Incremental learning

Instead of presenting all input at once, as required by the form of NMF introduced by ? (?), an incremental (adaptive) version of NMF was developed to mirror learning in a more plausible manner by ? (?). Adaptive NMF introduces an additional parameter: γ, which represents the weight of previous updates. The above-described process is adjusted as follows to process an input vector V from all inputs $\mathbf{V}$.

With the t's utterance in a sequence of T utterances in $\mathbf{V}$:

$$\mathbf{W}^t_{ik} \leftarrow \mathbf{W}^t_{ik} \sum_j H^t_{kj} \left(\frac{V}{\mathbf{W}H}\right)^t_{ij} + \gamma\kappa, \quad \text{with } \kappa = \mathbf{W}^{t-1}_{ik} \left(\frac{V}{\mathbf{W}H}\right)^{t-1}_{ij} H$$

$$\text{Normalise: } \sum_i \mathbf{W}^t_{ik} = 1$$

$$H_{kj} \leftarrow H_{kj} \sum i \mathbf{W}_{ik} \left(\frac{V}{\mathbf{W}H}\right)_{ij}$$

$$\text{Normalise: } \sum_i H_{ik} = 1$$

$\mathbf{W}^0$ (at the beginning of learning) and H (for each new utterance) are initialised with small random numbers using the MatLab function *rand*(), which returns a matrix containing pseudorandom values drawn from the standard uniform distribution on the interval (0,1).

Equalising the contributions of the acoustic and meaning subvectors

Since the meaning part of an input vector v_m comprises a much smaller number of coefficients (equal to the number of keywords in an experiment) than the acoustic part v_a (about 200 non-zero coefficients for the MFCC, Δ and $\Delta\Delta$ co-occurrences), the contribution of v_m to the distance function is multiplied with a weight factor, which is fixed to 100.

Testing

To test the model, new acoustic input v_a is approximated using only the acoustic-encoding part of the memory: $\mathbf{W}_a$, with the KL as cost function

(see equation **??**).

$$v_a \approx (\mathbf{W}_a \cdot \hat{h}) \tag{9}$$

$\hat{h}$ is obtained by using the lower two expressions in Eq. (**??**).

We assess model performance based on the approximated meaning information of a test utterance, which is obtained using the weights from the acoustic decoding step in equation **??**.

$$\hat{v}_m \approx (\mathbf{W}_m \cdot \hat{h}) \tag{10}$$

Simulated listening preferences

Listening preferences for sentences that contain a known word over sentences that do not contain a known keyword are computed. In this case, NMF is used to learn the matrix $\mathbf{W}$ from input vectors V which are comprised of an acoustic part v_a and a meaning part v_m. During test the acoustic sub-vector v_a^u of an utterance u is used to obtain the weight vector $\hat{h}^u$ by means of (**??**), which is then used to compute the meaning sub-vector $\hat{v}_m^u$. In the paper a distinction is made between *matching* and *recognition*. The *matching* score M^s for a sentence s is defined as

Matching: $M^u = \max_i \hat{v}_{m_i}^u$ for any m_i^u.

The *recognition* score R^u for utterance u is defined as the activation of the keyword that is present in utterance u.

Both M^u and R^u hold for utterance that either contain learned keywords

or not. In the experiments the preference values are summed over 20 test utterances for each keyword, measured at 10 points during the learning process. With p_{known}^{tk} the score for a test utterance k at testing moment t that contains a learned keyword, and $p_{unknown}^{tk}$ for the corresponding test utterance that does not contain a known keyword, and using the same expression for *matching* and *recognition* scores, the final preference score is obtained from

$$pref = \sum_{t}^{10} \sum_{k}^{20} p_{known}^{tk} - \frac{1}{3} \times \sum_{t}^{30} \sum_{k}^{20} p_{unknown}^{tk}$$

To account for the fact that three foils are matched to each target word, the sums over test sentences are divided by 3 for unknown words.
